# Falla Cardíaca con Fracción de Eyección Preservada: Un Problema de la Cardiología Contemporánea

**DOI:** 10.47487/apcyccv.v1i2.53

**Published:** 2020-06-29

**Authors:** Clara Saldarriaga-Giraldo, Cristhian Ramírez-Ramos, Catalina Gallego, Gustavo Castilla-Agudelo, Mateo Aranzazu-Uribe, Santiago Saldarriaga-Betancur

**Affiliations:** 1 Departamento de Cardiología Clínica y Falla Cardíaca, Clínica CardioVID y Universidad Pontificia Bolivariana. Docente de Cardiología Universidad de Antioquia. Medellín, Colombia. Médica internista, cardióloga y especialista en falla cardíaca. Universidad Pontificia Bolivariana Departamento de Cardiología Clínica y Falla Cardíaca Clínica CardioVID y Universidad Pontificia Bolivariana Medellín Colombia; 2 Departamento de Cardiología Clínica, Clínica CardioVID y Universidad Pontificia Bolivariana. Medellín, Colombia. Médico internista, fellow de Cardiología. Universidad Pontificia Bolivariana Departamento de Cardiología Clínica Clínica CardioVID y Universidad Pontificia Bolivariana Medellín Colombia; 3 Departamento de Cardiología Clínica y Cuidado Intensivo Cardiovascular Clínica CardioVID. Medellín, Colombia. Médica internista, cardióloga. Departamento de Cardiología Clínica y Cuidado Intensivo Cardiovascular Clínica CardioVID Medellín Colombia; 4 Departamento de Medicina Interna, Universidad Pontificia Bolivariana. Medellín, Colombia. Médico, residente de Medicina Interna. Universidad Pontificia Bolivariana Departamento de Medicina Interna Universidad Pontificia Bolivariana Medellín Colombia

**Keywords:** insuficiencia cardíaca, diástole, diagnóstico, heart failure, diastole, diagnosis

## Abstract

La falla cardíaca con fracción de eyección preservada (FCFep) constituye una entidad frecuente, sub-diagnosticada, que implica un gran reto diagnóstico y terapéutico. Datos actuales reportan una tendencia al incremento en su prevalencia, incluso por encima de la falla cardíaca con fracción de eyección reducida (FCFer). La fisiopatología es compleja e implica la contribución de múltiples factores como la interrelación genética, condiciones del estilo de vida y alta carga de condiciones crónicas (cardíacas y no cardíacas); lo cual lleva a la remodelación, mal adaptación y rigidez cardíaca, expresándose de manera tardía en síntomas como disnea, intolerancia al ejercicio y fatiga. Aunque la mortalidad y la tasa de supervivencia acumulada para pacientes con FCFep es tan igual como la de los pacientes con FCFer, ningún agente terapéutico ha mostrado de manera global efectividad en este tipo de pacientes, por lo que futuras propuestas arriesgan por un enfoque más individualizado en base al fenotipo de cada paciente.

## Abstract (en)

Heart failure with preserved ejection fraction (HFpEF) is a frequent and overlooked medical condition that represents a great challenge for diagnosis and treatment. Current data shows a temporal trend towards a higher prevalence of HFpEF, even above heart failure with reduced ejection fraction (HFrEF). The pathophysiology of HFpEF is heterogeneous and involves several factors such as genetics, lifestyle, and cardiac and non-cardiac comorbidities. These factors result in remodeling, maladaptation and cardiac stiffness, that later on cause dyspnea, exercise intolerance, and fatigue. Although the mortality outcome of HFpEF is as high as HFrEF, no specific therapy has demonstrated overall benefit in these patients; which is why future therapies will bet on an individualized approach according to the patients phenotype.

## Introducción

Se estima que aproximadamente 6.5 millones de personas en Estados Unidos tienen falla cardíaca y se espera que para el año 2030 se incremente en un 46%.^1^^)^ La mitad de estas personas tiene falla cardíaca con fracción de eyección preservada (FCFep). ^(^^2^^)^ A pesar de que el valor de la fracción de eyección del ventrículo izquierdo (FEVI), usada para definir este grupo, ha variado en los diferentes estudios, las ultimas guías restringen esta definición a pacientes con FEVI > 50%. Esto es importante pues la FEVI ha emergido como marcador fenotípico que indica el mecanismo fisiopatológico^3^^)^ y lo más importante: la respuesta terapéutica. ^(^^4^^)^ Se espera que la FCFep sea el fenotipo más prevalente de la falla cardíaca, con un aumento en la prevalencia de 1% al año; ^(^^5^^)^ y aunque esta entidad tiene una tasa de mortalidad similar a la falla cardíaca con FEVI reducida (FCFer) y comparte los mismos signos y síntomas (disnea, intolerancia al ejercicio y congestión), ^(^^6^^)^ nuestro entendimiento parcial de los mecanismos subyacentes de la enfermedad ha limitado y se ha convertido en una barrera no solo para el tratamiento sino también para una diagnóstico temprano y confiable. A la fecha, los fármacos utilizados en personas con FCFer, y que son efectivos en disminuir hospitalizaciones y mortalidad, han fallado por mostrar un beneficio en personas con FCFep seguramente siendo un reflejo de la heterogeneidad de esta condición. ^(^^7^^)^

## Epidemiología

### Incidencia

En el 2015, Gerber *et al.* ^(^^8^^)^ reportaron la incidencia de falla cardíaca específica para edad y sexo en Olmsted-Minnesota. La incidencia global de la enfermedad declinó de 315.8 por 100.000 habitantes en el año 2000 a 219.3 por 100.000 en el 2010, correspondiendo a un 37.5% de disminución en el periodo del estudio. El declive fue mayor para FCFer que para la FCFep (-45% y -28% respectivamente); sin embargo, la proporción de casos nuevos para FCFep pasó de 47.8% en el 2000-2003 a 56.9% en 2004-2007 y 52.3% en 2008-2010. 

Ho *et al.* ^(^^7^^)^ reportaron los resultados de 3 estudios de cohortes incluyendo el estudio de Framingham, el estudio de prevención de la enfermedad renal y vascular en estadio final (PREVEND por sus siglas en ingles Prevention of Renal and Vascular End-Stage Disease) y el estudio de salud cardiovascular (CHS por sus siglas Cardiovascular Health Study). Los 3 involucraron poblaciones con diferentes características basales como la edad, por lo que la incidencia acumulada de la enfermedad de manera global y la proporción de pacientes con FCFep varió en relación con esta característica; la incidencia más alta se documentó en la cohorte CHS (53%; edad media basal 73 años), la más baja en la cohorte PREVEND (37%; edad media basal 49 años) y una incidencia intermedia para la cohorte de Framingham (46%; edad media basal de 58 años). ^(^^9^

### Prevalencia

Un único estudio que proporciona estimaciones de la prevalencia de falla cardíaca por edad y sexo es la de una cohorte comunitaria. ^(^^10^^)^ En esta comunidad del sureste europeo, para ambos tipos de falla cardíaca la prevalencia se incrementó con la edad, pero a cualquier edad la prevalencia de FCFep fue mayor en mujeres que en hombres y su crecimiento fue más rápido con la edad comparado con la FCFer. Este y otros estudios han reportado un mayor porcentaje de mujeres que hombres en esta forma de la enfermedad. ^(^^9^

## Fisiopatología

A nivel macroscópico, la FCFep se distingue de la FCFer en virtud del remodelado concéntrico del ventrículo izquierdo, donde hay un incremento en el grosor y de la masa de la cavidad, lo que genera hipertrofia. ^(^^11^^)^ El siguiente paso es la aparición del marcador fisiopatológico distintivo: la rigidez tisular aumentada de la cavidad. Como resultado de este, se altera la relajación y aumentan las presiones de llenado al final de la diástole. Para mantener el volumen latido y una mecánica eficiente, se incrementa el rendimiento sistólico para así mantener la fracción de eyección constante durante el reposo. ^(^^12^^)^ El llenado diastólico ventricular también cambia: en corazones sanos, a medida que la cavidad se relaja en la fase temprana de la diástole, se crea una presión negativa que succiona la sangre de la aurícula izquierda y se produce el 70-80% del llenado del ventrículo; el restante 20-30% se produce en la contracción auricular. Con el empeoramiento progresivo de la disfunción diastólica, el llenado durante la diástole temprana disminuye y la presión aumenta en la aurícula lo que genera dilatación de esta cavidad y el llenado se produce por una presión más positiva que negativa. Los cambios estructurales y funcionales descritos toman varios años en desarrollarse y cuando el paciente se diagnostica en base a los signos y síntomas, usualmente está en una etapa avanzada de la enfermedad. ^(^^13^^)^

**Figura 1:**
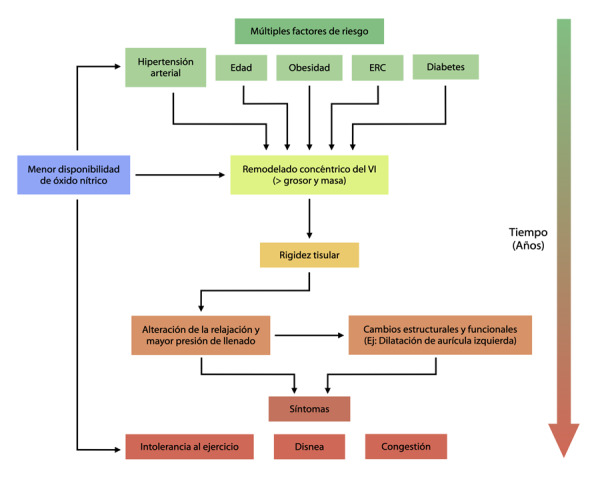
Fisiopatología de la Falla Cardíaca con Fracción de Eyección Preservada. ERC: Enfermedad renal crónica

El paradigma de entendimiento actual soporta el papel de la inflamación sistémica producto de las comorbilidades como un iniciador clave de la enfermedad. (Figura 1) En este sentido, el primer estadio es la inflamación del endotelio que reduce la biodisponibilidad del oxido nítrico, un factor clave regulador de la vasodilatación y relajación del músculo liso. Su acción es mediada por la vía de señalización oxido nítrico - guanilato ciclasa soluble - monofosfato de guanosina cíclico, conocida en la actualidad como una vía clave en la regulación de la función cardíaca, mediando efectos a nivel inotrópico, cronotrópico y lusotrópico. ^(^^14^^)^

En pacientes con falla cardíaca, los niveles bajos de óxido nítrico reducen la cantidad de monofosfato de guanosina cíclico y la actividad de la proteína kinasa G, lo que promueve la hipertrofia y el retraso en la relajación miocárdica. Su impacto directo en la función cardíaca se agrava por los efectos de la molécula (oxido nítrico) en la circulación sistémica lo que altera la precarga y postcarga. ^(^^15^^)^ Otra consecuencia de la disfunción en la circulación sistémica se aprecia a nivel del músculo esquelético, donde la disfunción del endotelio genera la intolerancia al ejercicio vista de manera común en estos pacientes. ^(^^16^^)^ La microcirculación coronaria también se compromete por la inflamación endotelial, algo constatado en biopsias miocárdicas y lo que explica el dolor torácico que se produce por la disminución de la perfusión y las alteraciones microvasculares. ^(^^17^^)^ Junto con estos cambios la inflamación cardíaca inicia procesos fibróticos, los cuales también se han evidenciado en especímenes miocárdicos de pacientes con FCFep. ^(^^18^^)^ Como se nota, esta condición se conoce en la actualidad como un síndrome sistémico más que como una condición cardíaca aislada, algo que seguramente podrá guiarnos a encontrar un tratamiento efectivo.

## Comorbilidades

Respecto al perfil clínico, suelen ser pacientes mayores (alrededor de 75 años), mujeres (55-73%) y a tener múltiples comorbilidades como hipertensión (75%), obesidad/sobrepeso (>80%), diabetes (40%) y enfermedad renal (25-50%).^19^^)^ Estas patologías son consideradas factores de riesgo mayores de la enfermedad. A pesar de las asociaciones conocidas de la FCFep y estas condiciones, estos pacientes no son tamizados para enfermedad cardíaca hasta que tienen síntomas, lo cual es a menudo tardío en la evolución. Las pacientes con diabetes tienen 10 veces incremento de riesgo de muerte y la supervivencia a 5 años es del 15.5% cuando tienen síntomas de falla cardíaca. ^(^^20^^)^ Los pacientes obesos tampoco reciben atención hasta que aparecen los síntomas de falla cardíaca, a pesar de que sabemos que la obesidad incrementa la rigidez aórtica y la carga miocárdica, lo que aumenta la hipertrofia. Un estudio de pacientes con FCFep demostró que los pacientes con obesidad abdominal tenían un resultado peor en cuanto a mortalidad por todas las causas comparado con los no obesos, ^(^^21^^)^ lo que resalta la importancia de esta asociación. 

## Diagnóstico

**Figura 2.**
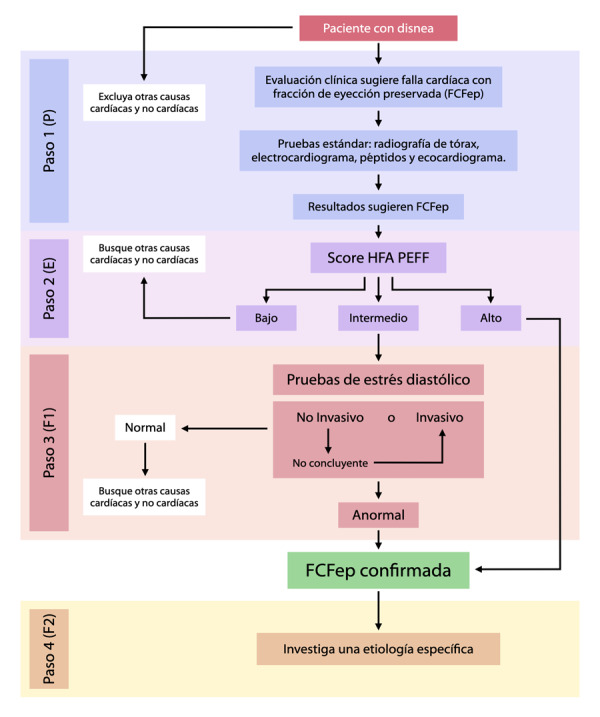
Abordaje diagnóstico en la Falla Cardíaca con Fracción de Eyección Preservada. Adaptado de Pieske B, Tschöpe C, de Boer R et al. How to diagnose heart failure with preserved ejection fraction: the HFA-PEFF diagnostic algorithm: a consensus recommendation from the Heart Failure Association (HFA) of the European Society of Cardiology (ESC). Eur Heart J. 2019;40(40):3297-317.

Con el transcurrir del tiempo y con los avances en la investigación de los aspectos fisiopatológicos de la enfermedad, se ha intentado mejorar la forma de realizar el abordaje de los pacientes con FCFep. A continuación, se menciona el abordaje paso a paso recomendado por la Asociación de Insuficiencia Cardíaca de la Sociedad Europea de Cardiología, ^(^^22^^)^ que incluye una valoración inicial y el uso de péptidos y parámetros ecocardiográficos con la novedad de la prueba de estrés diastólico, y el énfasis en llegar siempre a la etiología para cada caso diagnosticado. (Figura 2)

### PASO 1 (P): Evaluación Pre-test

Requiere una historia clínica y demográfica, electrocardiograma, pruebas sanguíneas básicas, ecocardiografía estándar, además de estudios en busca de arritmias, isquemia, enfermedad pulmonar en base individual de cada paciente. Los péptidos se pueden obtener si están disponibles. ^(^^22^^)^

*Signos y síntomas:* La disnea de ejercicio (New York Heart Association Clase II o III) es altamente sensible para el diagnóstico de falla cardíaca pero sólo moderadamente específica (alrededor del 50%) para una causa cardíaca. ^(^^22^

*Electrocardiograma:* Se pueden encontrar cambios compatibles con hipertrofia o crecimiento auricular, ambos con bajo nivel de sensibilidad. Lo más importante es detectar fibrilación auricular, un fuerte predictor de la enfermedad. ^(^^22^^)^

*Pruebas de laboratorio recomendadas:* Se debe pedir un perfil hemático completo, con conteo celular diferencial, electrolitos séricos, función tiroidea, así como ferrocinética. Debe recordarse que los niveles bajos de hemoglobina agravan los síntomas de la enfermedad. ^(^^22^

*Péptidos natriuréticos:* Los valores de NT-proBNP menores a 125 pg/ml o BNP por debajo de 35 pg/ml, tienen un valor predictivo negativo de 95-99% para descartar que los síntomas sean de origen cardiovascular; sin embargo, aproximadamente el 20% de los pacientes tienen niveles por debajo de estos puntos de corte y presentan diagnóstico de FCFep por pruebas invasivas. Por lo tanto, resaltamos que un solo parámetro no sirve para incluir o excluir la enfermedad. ^(^^22^^)^

*Ecocardiograma estándar:* Este examen se realiza en pacientes con disnea en quienes se sospecha de falla cardíaca. Sin embargo, si el paciente tiene menos de 70 años en hombres o de 60 en mujeres, no tiene historia de obesidad o alteraciones de peso, síndrome metabólico, diabetes mellitus, fibrilación auricular o hipertensión arterial, tiene un electrocardiograma normal, con buen nivel actividad física tolerancia al ejercicio, y los valores medidos de los péptidos están por debajo de los previamente citados, no tendría indicación realizarse esta prueba. El ecocardiograma ayuda a excluir otros diagnósticos como hipertensión pulmonar, enfermedad valvular, FCFer o derrame pericárdico. Los hallazgos que soportan el diagnóstico de FCFep son un ventrículo izquierdo no dilatado con FEVI normal, remodelado concéntrico o hipertrofia y dilatación de la aurícula izquierda; sin embargo, la ausencia de anormalidades estructurales no excluye esta condición. 

### PASO 2 (E): Score Ecocardiográfico (Valoración Morfológica-Funcional) y de Péptidos Natriuréticos

**Figura 3:**
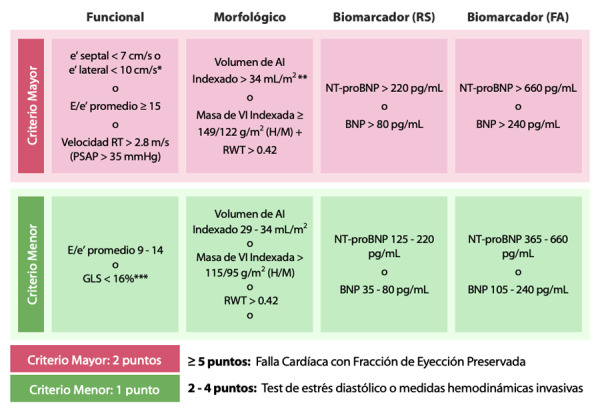
Paso 2(E). Valores para tener en cuenta en el cálculo de la escala.

Se recomiendan criterios mayores (mayor especificidad) y menores (mayor sensibilidad) con diferentes puntos de corte. (Figura 3) Algunos parámetros ecográficos a tener en cuenta son:

Onda e´: Refleja el estiramiento del ventrículo izquierdo. 

Radio E/e’: Correlaciona con la rigidez y fibrosis de ventrículo izquierdo. 

Strain longitudinal global: Pacientes con anormalidades de esta medición tienen más riesgo de muerte cardiovascular, paro cardiorrespiratorio y hospitalizaciones. Este parámetro se ha correlacionado con los niveles séricos de los péptidos natriuréticos y además con las mediciones de rigidez realizadas de manera invasiva. 

Volumen indexado de la aurícula izquierda (LAVI): Es un fuerte predictor de eventos cerebrovasculares, fibrilación auricular, falla cardíaca y mortabilidad global. 

Los criterios no son aditivos en una misma categoría (Ejemplo: si cumple 1 mayor y 1 menor en categoría funcional, sólo obtiene 2 puntos). * Para mayores de 75 años los valores son e´ septal <5 cms/s y e´ lateral < 7 cms/s ** Para pacientes con fibrilación auricular se considera > 40 mL/m^2^ como criterio mayor y entre 34 y 40 mL/m^2^ como criterio menor. *** Strain longitudinal global menos negativo que -16%. RS: ritmo sinusal; FA: fibrilación auricular; RT: regurgitación tricuspídea; GLS: strain longitudinal global (por sus siglas en inglés); AI: aurícula izquierda; VI: ventrículo izquierdo; H: hombre; M: mujer; RWT: grosor relativo de la pared (por sus siglas en inglés).

Adaptado de Pieske B, Tschöpe C, de Boer R et al. How to diagnose heart failure with preserved ejection fraction: the HFA-PEFF diagnostic algorithm: a consensus recommendation from the Heart Failure Association (HFA) of the European Society of Cardiology (ESC). Eur Heart J. 2019;40(40):3297-317.

Con valores mayores o iguales de 5, se considera que el paciente tiene falla cardíaca. Si el puntaje es menor o igual a 1, es poco probable, y valores entre 2 y 4 requieren continuar en el paso 3 del algoritmo. ^(^^22^^)^ Varias características de un mismo dominio no son aditivas.

### Paso 3 (F1): Pruebas Funcionales

*Ecocardiograma de estrés:* La relación E/e´ así como la velocidad de regurgitación tricuspídea se han estudiado en la prueba de estrés diastólico, pues estos reflejan un incremento en la presión en cuña capilar pulmonar media y en la presión sistólica de arteria pulmonar, respectivamente. Fisiopatológicamente las personas con FCFep tienen alteración de la *compliance* ventricular, una relajación diastólica temprana anormal, así como una incapacidad para incrementar la succión en la diástole que se refleja en un inadecuado incremento del volumen latido y del gasto cardíaco en el ejercicio. Estas alteraciones explican los síntomas desarrollados con el esfuerzo físico y se pueden estimar de manera indirecta por un cambio, tanto en la regurgitación tricuspídea y en la relación E/e´.^16^^)^

Valores de E/e´ mayores o iguales a 15 durante el ejercicio máximo con o sin una velocidad de regurgitación tricuspídea > 3.4 m/s son considerados anormales. ^(^^22^^)^ Solo el cambio en la regurgitación no debe usarse pues este valor puede reflejar la respuesta hiperdinámica al ejercicio. Si cumple los dos criterios, se suman 3 puntos al cálculo realizado previamente; si solo se cumple el radio E/e´ se adicionan 2 puntos. Si al realizar estas valoraciones adicionales, el paciente suma 5 o más puntos se confirma el diagnóstico de la enfermedad. Si el puntaje permanece menor de 5, se recomienda realizar una prueba invasiva. 

*Limitaciones:* En el 10% de los sujetos durante ejercicio submáximo y 20% en el máximo nivel no se puede realizar mediciones del radio E/e´, siendo esta limitación casi del 50% para la velocidad de regurgitación tricúspidea. Por otro lado, la tasa de falsos positivos puede llegar a 20% en controles sanos. ^(^^22^^)^

*Pruebas invasivas en reposo y ejercicio:* Si la presión de fin de diástole de ventrículo izquierdo (PFDVI) es mayor o igual de 16 mmHg, se confirma como causa una FCFep. Si las mediciones del ventrículo izquierdo no están disponi-bles, se puede considerar el cateterismo derecho; la presión en cuña capilar pulmonar media (mPCWP) mayor o igual a 15 mmHg es evidencia definitiva de FCFEp (en el contexto de FEVI > 50%). Si estos valores son normales, no se excluye la enfermedad debido a que, como se mencionó, las alteraciones en algunos pacientes solo se presentan durante el ejercicio. Por ello, ante valores normales en reposo en pacientes con sospecha de falla cardíaca y escala intermedia, o si las pruebas de estrés fueron no diagnósticas, se recomienda realizar un cateterismo derecho en ejercicio dependiendo de la disponibilidad. Los puntos de corte en sujetos sanos son < 25 mmHg para PDFVI y < 20-23 mmHg para PCWP. ^(^^22^^)^

### Paso 4 (F2): Etiología Final

Teniendo en cuenta las múltiples causas que pueden manifestarse como una FCFep, debe asegurarse que no exista una causa específica de FCFep distinta a la relacionada a hipertensión, edad y obesidad. Para ello, debe considerarse las siguientes pruebas: resonancia magnética cardiaca, prueba de esfuerzo, gammagrafía de tecnecio-dipiridamol (TC-DPD), PET-CT e incluso biopsia miocárdica. Estos últimos se realizan en casos seleccionados dependiendo del contexto clínico y los hallazgos de otros estudios iniciales, con el objetivo de identificar enfermedades como Fabry o la amiloidosis para las cuales se dispone de tratamiento específico que puede modificar el curso de la enfermedad. El estudio que ha mostrado gran utilidad en el diagnóstico de muchas causas de falla cardíaca es la resonancia magnética. ^(^^22^

## Tratamiento

Varios ensayos clínicos con medicamentos como los inhibidores de la enzima convertidora de angiotensina (PEP-CHF trial^23^^)^ con perindopril), antagonistas del receptor de angiotensina (CHARM-preserved^24^^)^ con candesartán, I-PRESERVE^25^^)^ con irbesartán), betabloqueadores (J-DHF trial^26^^)^ con carvedilol, ELANDD trial^27^^)^ con nebivolol) fallaron en demostrar beneficio alguno en la FCFep. En el ensayo clínico TOPCAT se aleatorizaron 3445 pacientes a recibir espironolactona (15-45 mg día) o placebo; aquí se encontró un resultado neutro para el resultado primario (muerte cardiovascular, hospitalización por falla cardíaca, paro cardíaco abortado). Sin embargo, en el grupo de espironolactona se encontró evidencia en disminución de las hospitalizaciones por falla cardíaca (12% vs. 14.2%, HR 0.83, IC 95% (0.69-0.99), p 0.04). ^(^^28^^)^ Análisis posteriores del estudio generaron controversia pues los pacientes eran muy heterogéneos con evidencia de falta de disfunción diastólica en 30% de la población^29^^)^ y además la observación de resultados distintos para la población de las Américas (Estados Unidos, Argentina, Brasil y Canadá) con respecto a la de Rusia. ^(^^30^^)^ A raíz de esta discrepancia demográfica en el estudio, se buscó analizar el suero recolectado de estas últimas poblaciones, lo que demostró mayores tasas de niveles no detectables de canrenona en Rusia con respecto a las Américas, sugiriendo falta de adherencia al protocolo o ausencia de toma de medicamento en este país. ^(^^31^^)^ Asimismo, en un análisis de subgrupos del TOPCAT, se observó que aquellos con niveles elevados de péptidos natriuréticos tenían más riesgo del evento primario, pero esto no guardaba asociación de beneficio con el uso de espironolactona, que sí se observó en el grupo con menor nivel de péptidos natriuréticos. ^(^^28^

Por último, el estudio PARAGON-HF aleatorizó 4822 pacientes (2407 en el grupo sacubitril - valsartán y 2389 pacientes a recibir valsartán) con el objetivo de analizar su impacto en hospitalizaciones y muerte cardiovascular. Los resultados no mostraron beneficio de la terapia de sacubitril - valsartán, salvo en el subgrupo de mujeres y pacientes con fracción de eyección ≤ 57%.^32^^)^

Es así que, hasta el último reporte de la guía de falla cardíaca del año 2016 de la Sociedad Europea de Cardiología, el manejo actual recomendado está dirigido al manejo de las patologías de base del paciente y al alivio de la congestión con diuréticos cuando esté presente. ^(^^33^^)^ Sin embargo, por la alteración fisiopatológica en relación con las condiciones de base, la alteración en el metabolismo de las células miocárdicas y los resultados tan contundentes del estudio DAPA-HF^34^^)^ de pacientes con fracción de eyección reducida, se encuentran en curso múltiples ensayos clínicos intentando probar si los inhibidores del transportador sodio glucosa - 2 (ISGLT-2) son la respuesta que aún buscamos en el manejo farmacológico que modifique el curso de esta enfermedad. Por otro lado, datos iniciales del uso de vericiguat, un estimulador de la guanilato ciclasa soluble, en el estudio SOCRATES-PRESERVED, mostraron mejoría significativa en el estado de salud de los pacientes incluidos, medidos por la escala de Kansas (KCCQ). ^(^^35^^)^ Desafortunadamente, los resultados preliminares de este grupo de medicamentos presentados en el último congreso virtual de falla cardíaca de la Sociedad Europea de Cardiología, no pudieron demostrar beneficio en mejoría de la calidad de vida. 

## Pronóstico

En general, la mortalidad es mayor para las personas con FCFep comparado con controles sin la enfermedad pareados por sexo y edad. ^(^^36^^)^ La mortalidad hospitalaria varía entre 2.4%^5^^)^ y 4.9%^37^^)^ en los estudios observacionales, siendo levemente mayor a 30 días (5%)^36^^)^ y a los 60-90 días (9.5%).^38^^)^ La mortalidad al año es de 20% a 29%^(8, 36, 32)^ siendo mayor para los trabajos que incluían solo pacientes hospitalizados. A 5 años la mitad de los pacientes han muerto, con mortalidades reportadas entre 53% y 74%.^8^^,^^39^^)^ Los pacientes con mayor carga de enfermedades de base son los que tienen riesgo mayor de muerte. 

Las causas de muerte son en general de etiología cardiovascular; sin embargo, las condiciones no cardiovasculares pueden explicar hasta la mitad de los casos en algunos estudios observacionales. La muerte súbita es menos común que en los pacientes con FCFer, pero genera un porcentaje no despreciable de 25% de manera global y 40% de las muertes de causa cardiovascular. ^(^^40^^)^

Las rehospitalizaciones son comunes en estos pacientes tanto por causas cardíacas como no cardíacas (evento cerebrovascular, embolismo pulmonar, etc.), con tasas de rehospitalización por causas cardíacas de 20% dentro de 30 días del alta y mayor de 50% al año. ^(^^41^^)^ El 90% se relaciona a congestión persistente que altera cada vez más la función cardiovascular.

## Conclusiones

La FCFep es una entidad altamente prevalente y sin embargo, muchas veces subdiagnosticada. Es un desorden heterogéneo ocasionado por la combinación de muchas condiciones (cardíacas y no cardíacas) que generan inflamación crónica con alteración de la función diastólica y la estructura cardíaca (miopatía atrial y ventricular). En base al pronóstico que esta conlleva en calidad y cantidad de vida es que se debe hacer un abordaje paso a paso para identificar al posible paciente con FCFep y la etiología causante (cardiomiopatía, valvular, isquémico, hipertensivo, etc). Por último, si bien existen varios estudios en curso para disminuir rehospitalizaciones, mejorar calidad de vida y disminuir mortalidad; hasta el momento el tratamiento queda relegado al manejo integral de las comorbilidades, con estrategias y esperanzas futuras que buscan un abordaje específico para cada fenotipo particular de esta enfermedad.

## Financiación:

Ninguna

## References

[1] Heidenreich P, Albert N, Allen L (2013). Forecasting the impact of heart failure in the United States a policy statement from the American Heart Association. Circ Heart Fail.

[2] Go A, Mozaffarian D, Roger V (2014). Heart disease and stroke statistics--2014 update a report from the American Heart Association. Circulation.

[3] Borlaug B, Redfield M (2011). Diastolic and systolic heart failure are distinct phenotypes within the heart failure spectrum. Circulation.

[4] Redfield M (2016). Heart Failure with Preserved Ejection Fraction. N Engl J Med.

[5] Steinberg B, Zhao X, Heidenreich P (2012). Trends in patients hospitalized with heart failure and preserved left ventricular ejection fraction prevalence, therapies, and outcomes. Circulation.

[6] Borlaug B, Paulus W (2011). Heart failure with preserved ejection fraction pathophysiology, diagnosis, and treatment. Eur Heart J.

[7] Ho J, Enserro D, Brouwers F (2016). Predicting Heart Failure With Preserved and Reduced Ejection Fraction: The International on Heart Failure Subtypes. Circ Heart Fail.

[8] Gerber Y, Weston S, Redfield M (2015). A contemporary appraisal of the heart failure epidemic in Olmsted County, Minnesota, 2000 to 2010. JAMA Intern Med.

[9] Dunlay S, Roger V, Redfield M (2017). Epidemiology of heart failure with preserved ejection fraction. Nat Rev Cardiol.

[10] Ceia F, Fonseca C, Mota T (2002). Prevalence of chronic heart failure in Southwestern Europe the EPICA study. Eur J Heart Fail.

[11] Mohammed S, Borlaug B, Roger V (2012). Comorbidity and ventricular and vascular structure and function in heart failure with preserved ejection fraction a community-based study. Circ Heart Fail.

[12] Gladden J, Linke W, Redfield M (2014). Heart failure with preserved ejection fraction. Pflugers Arch.

[13] Loai S, Cheng H (2020). Heart failure with preserved ejection fraction the missing pieces in diagnostic imaging. Heart Fail Rev.

[14] Lim S, Lam C, Segers V (2015). Cardiac endothelium-myocyte interaction clinical opportunities for new heart failure therapies regardless of ejection fraction. Eur Heart J.

[15] Van Heerebeek L, Hamdani N, Falcao-Pires I (2012). Low myocardial protein kinase G activity in heart failure with preserved ejection fraction. Circulation.

[16] Borlaug B, Olson T, Lam C (2010). Global cardiovascular reserve dysfunction in heart failure with preserved ejection fraction. J Am Coll Cardiol.

[17] Paulus W, Tschope C (2013). A novel paradigm for heart failure with preserved ejection fraction comorbidities drive myocardial dysfunction and remodeling through coronary microvascular endothelial inflammation. J Am Coll Cardiol.

[18] Westermann D, Lindner D, Kasner M (2011). Cardiac inflammation contributes to changes in the extracellular matrix in patients with heart failure and normal ejection fraction. Circ Heart Fail.

[19] Campbell R, McMurray J (2014). Comorbidities and differential diagnosis in heart failure with preserved ejection fraction. Heart Fail Clin.

[20] Hippisley-Cox J, Coupland C (2016). Diabetes treatments and risk of heart failure, cardiovascular disease, and all cause mortality cohort study in primary care. BMJ.

[21] Tsujimoto T, Kajio H (2017). Abdominal Obesity Is Associated With an Increased Risk of All-Cause Mortality in Patients With HFpEF. J Am Coll Cardiol.

[22] Pieske B, Tschope C, de Boer R (2019). How to diagnose heart failure with preserved ejection fraction the HFA-PEFF diagnostic algorithm: a consensus recommendation from the Heart Failure Association (HFA) of the European Society of Cardiology (ESC). Eur Heart J.

[23] Cleland J, Tendera M, Adamus J (2006). The perindopril in elderly people with chronic heart failure (PEP-CHF) study. Eur Heart J.

[24] Yusuf S, Pfeffer M, Swedberg K (2003). Effects of candesartan in patients with chronic heart failure and preserved left-ventricular ejection fraction the CHARM-Preserved Trial. Lancet Lond Engl.

[25] Massie B, Carson P, McMurray J (2008). Irbesartan in patients with heart failure and preserved ejection fraction. N Engl J Med.

[26] Yamamoto K, Origasa H, Hori M, J-DHF Investigators (2013). Effects of carvedilol on heart failure with preserved ejection fraction the Japanese Diastolic Heart Failure Study (J-DHF). Eur J Heart Fail.

[27] Conraads V, Metra M, Kamp O (2012). Effects of the long-term administration of nebivolol on the clinical symptoms, exercise capacity, and left ventricular function of patients with diastolic dysfunction results of the ELANDD study. Eur J Heart Fail.

[28] Pitt B, Pfeffer M, Assmann S (2014). Spironolactone for heart failure with preserved ejection fraction. N Engl J Med.

[29] Shah A, Shah S, Anand I (2014). Cardiac structure and function in heart failure with preserved ejection fraction baseline findings from the echocardiographic study of the Treatment of Preserved Cardiac Function Heart Failure with an Aldosterone Antagonist trial. Circ Heart Fail.

[30] Pfeffer M, Claggett B, Assmann S (2015). Regional variation in patients and outcomes in the Treatment of Preserved Cardiac Function Heart Failure With an Aldosterone Antagonist (TOPCAT) trial. Circulation.

[31] de Denus S, O'Meara E, Desai A (2017). Spironolactone Metabolites in TOPCAT - New Insights into Regional Variation. N Engl J Med.

[32] Solomon S, McMurray J, Anand I (2019). Angiotensin-Neprilysin Inhibition in Heart Failure with Preserved Ejection Fraction. N Engl J Med.

[33] Ponikowski P, Voors A, Anker S (2016). 2016 ESC Guidelines for the diagnosis and treatment of acute and chronic heart failure The Task Force for the diagnosis and treatment of acute and chronic heart failure of the European Society of Cardiology (ESC) Developed with the special contribution of the Heart Failure Association (HFA) of the ESC. Eur Heart J.

[34] McMurray J, Solomon S, Inzucchi S (2019). Dapagliflozin in Patients with Heart Failure and Reduced Ejection Fraction. N Engl J Med.

[35] Filippatos G, Maggioni A, Lam C (2017). Patient-reported outcomes in the SOluble guanylate Cyclase stimulatoR in heArT failurE patientS with PRESERVED ejection fraction (SOCRATES-PRESERVED) study. Eur J Heart Fail.

[36] Bhatia R, Tu J, Lee D (2006). Outcome of heart failure with preserved ejection fraction in a population-based study. N Engl J Med.

[37] Pérez de Isla L.Cañadas V.Contreras L et al (2009). Diastolic heart failure in the elderly in-hospital and long-term outcome after the first episode. Int J Cardiol.

[38] Fonarow G, Stough W, Abraham W (2007). Characteristics, treatments, and outcomes of patients with preserved systolic function hospitalized for heart failure a report from the OPTIMIZE-HF Registry. J Am Coll Cardiol.

[39] Owan T, Hodge D, Herges R (2006). Trends in prevalence and outcome of heart failure with preserved ejection fraction. N Engl J Med.

[40] Vaduganathan M, Patel R, Michel A (2017). Mode of Death in Heart Failure With Preserved Ejection Fraction. J Am Coll Cardiol.

[41] Cheng R, Cox M, Neely M (2014). Outcomes in patients with heart failure with preserved, borderline, and reduced ejection fraction in the Medicare population. Am Heart J.

